# Supplementary material for: Mapping the subcortical connectivity of the human default mode network
Source: Neuroimage. Author manuscript; Available in PMC 2022 Mar 24. (PMC8945548; doi:10.1016/j.neuroimage.2021.118758)
Supplement: 2 [file NIHMS1770958-supplement-2.pdf]

Supplementary Material

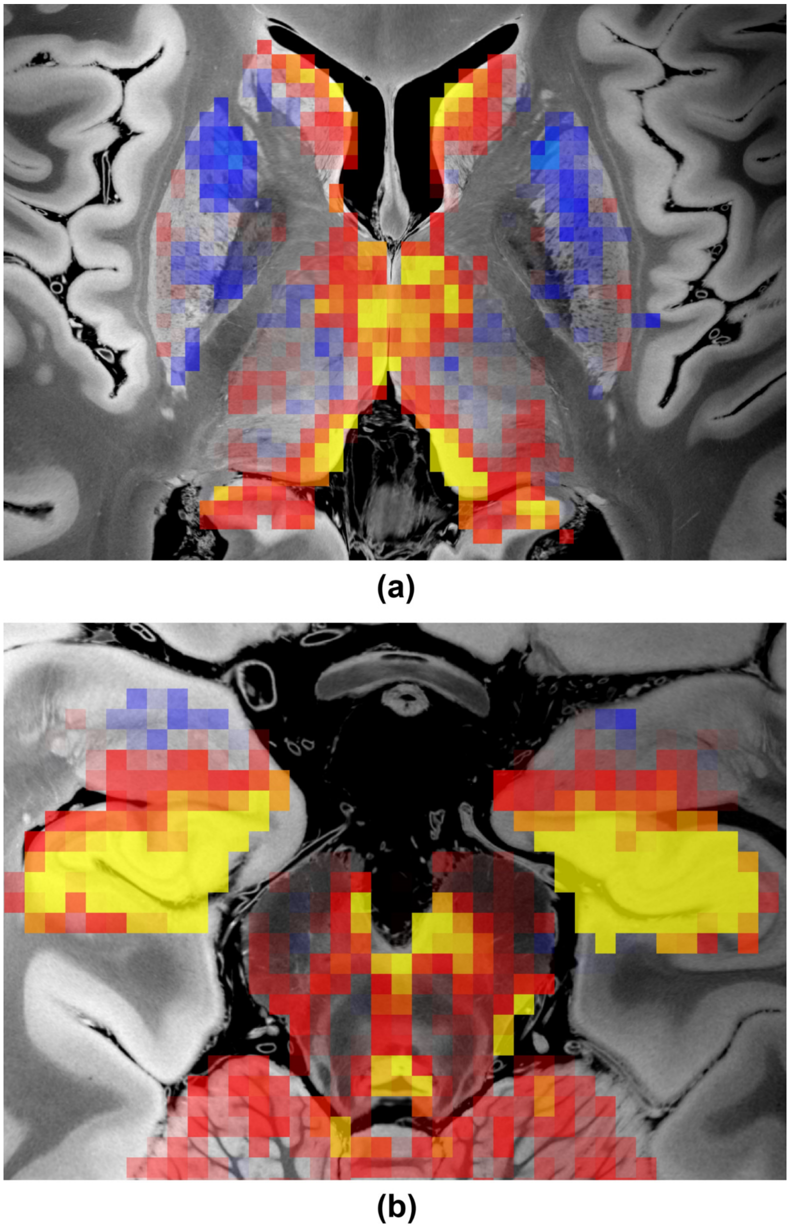

Fig. S1. Subcortical map of the default mode network obtained using the Nadam-accelerated SCAlable and Robust tensor decomposition method in the original resting-state functional MRI resolution. Representative images are shown in the axial plane at the level of the (a) mid-thalamus and (b) caudal midbrain.

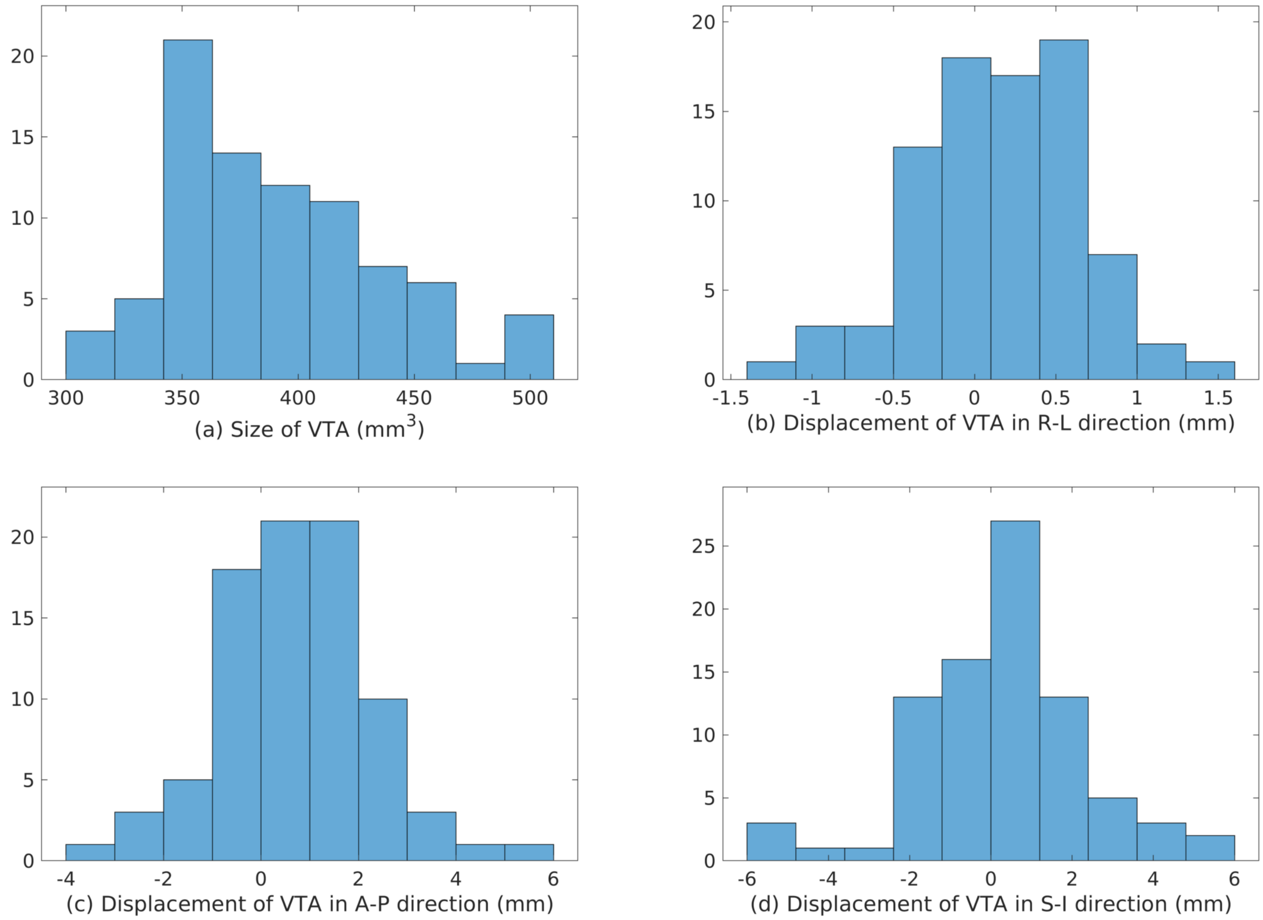

Fig. S2. Histograms of measures of the ventral tegmental area (VTA) in subject's individual space. (a) The volume of VTA; (b) Displacement of VTA during registration of subject to MNI space in Right-Left (R-L) direction; (b) Same as (b) but in Anterior-Posterior (A-P) direction; (d) Same as (b) but in Superior-Inferior (S-I) direction.
